# A mixture of feature experts approach for protein-protein interaction prediction

**DOI:** 10.1186/1471-2105-8-S10-S6

**Published:** 2007-12-21

**Authors:** Yanjun Qi, Judith Klein-Seetharaman, Ziv Bar-Joseph

**Affiliations:** 1 School of Computer Science, Carnegie Mellon University, Pittsburgh, PA 15213 USA; 2 Department of Structural Biology, University of Pittsburgh School of Medicine, Pittsburgh, PA 15260 USA

## Abstract

**Background:**

High-throughput methods can directly detect the set of interacting proteins in model species but the results are often incomplete and exhibit high false positive and false negative rates. A number of researchers have recently presented methods for integrating direct and indirect data for predicting interactions. These methods utilize a common classifier for all pairs. However, due to missing data and high redundancy among the features used, different protein pairs may benefit from different features based on the set of attributes available. In addition, in many cases it is hard to directly determine which of the data sources contributed to a prediction. This information is important for biologists using these predications in the design of new experiments.

**Results:**

To address these challenges we propose a Mixture-of-Feature-Experts method for protein-protein interaction prediction. We split the features into roughly homogeneous sets of feature experts. The individual experts use logistic regression and their scores are combined using another logistic regression. When combining the scores the weighting of each expert depends on the set of input attributes available for that pair. Thus, different experts will have different influence on the prediction depending on the available features.

**Conclusion:**

We applied our method to predict the set of interacting proteins in yeast and human cells. Our method improved upon the best previous methods for this task. In addition, the weighting of the experts provides means to evaluate the prediction based on the high scoring features.

## Background

Pair-wise protein-protein interactions (PPIs) are the building blocks of complexes and pathways which carry out different biological processes. Correctly identifying the set of interacting proteins in an organism is useful for deciphering the molecular mechanisms underlying biological functions and for assigning functions to unknown proteins based on their interacting partners. Even for model organisms such as yeast, most PPIs have not been discovered yet.

A number of high-throughput experimental approaches have been applied to determine the set of interacting proteins on a proteome-wide scale. These include the two-hybrid (Y2H) screens [1-4], which detect both transient and stable interactions and mass spectrometry methods, that are used to identify components of protein complexes [5,6]. However, both methods suffer from high false positive and false negative rates [7]. For instance in yeast, roughly 80,000 interactions have been predicted by various high-throughput methods, but only a small number (~2,400) are supported by more than one method. In addition to experiments that directly test for PPI, there are many indirect sources that may contain information about PPIs. For example, it has been shown that many interacting pairs are co-expressed [7] and expression of proteins found in the same complex is in some cases controlled by the same transcription factor(s) [8]. Sequence data has also been used to infer such interactions (for example by relying on domain-domain interactions and structure information [9]). Each of these datasets provides partial information about the interacting pairs. These findings suggest that direct data on protein interactions can be combined with indirect information to improve the success of protein interaction prediction.

Researchers have recently suggested a number of methods to predict protein interactions by combining both direct evidence and indirect information. Most studies have been carried out in yeast. Jansen et al. [10] combined multiple data sources using a Bayes classifier for PPI predictions in yeast. Lin et al. [12] compared Jansen's method with two other classifiers, Random Forest (RF) and Logistic Regression (LR) and found RF to be the best among them. Qi et al. [15] extended this comparison to include three more classifiers and additional 'gold standard' datasets. Their results confirmed that RF performs best among all classifiers for this task and have also indicated that Support Vector Machines (SVMs) are preforming very well on this task.

Zhang et al. [17] constructed a decision tree to predict co-complexed protein pairs by integrating genomic and proteomic data. Ben-Hur et al. [13] used kernel machines for this task. Yamanishi et al. [16] predicted pathway protein interactions using a variant of kernel canonical correlation analysis. Compared to yeast, human is more complex and there are fewer attempts at predicting human PPIs so far. Rhodes et al. [18] employed a sum of likelihood ratio scores strategy to predict human PPI confidence. Brown and Jurisica [19] derived a more reliable set of human PPIs using evolutionary information. All of the above methods were shown to improve the success of PPI prediction when compared to direct data alone. The improvements are not just from the perspective of predicting novel interactions but also for the purpose of stratifying the many candidate interactions by confidence. While useful, the above methods do not address two important problems in this domain. First, these classification methods estimate a set of parameters that are used for all input pairs. However, the existing biological datasets contain many missing values and highly correlated features. Thus, different protein pairs may benefit from using different feature sets. The second problem is that biologists who want to use these methods to design experiments cannot easily determine which of the features contributed to a resulting prediction. Since different researchers may have different opinions regarding the reliability of the various features, it is useful if the method can indicate, for every pair, which feature contributed the most to the classification result.

In this paper we address the above challenges using a Mixture-of-Feature-Experts (MFE) method. We divide the biological datasets into several groups. Each of the groups represents a specific data type and is used by a feature expert (classifier) to predict interactions. Results from all experts are combined such that the weight of each expert depends on the input sample and thus varies between input pairs. This weight can also indicate the importance of the features used by this expert for predicting a pair. We applied our method to predict PPIs in yeast and human. Using Precision vs. Recall curves and AUC scores we show that the MFE method improved upon traditional classification methods that were previously applied for predicting PPIs. For a specific Yeast pathway, the pheromone pathway, we show that it is possible to extract confidence information from the weight distribution, in addition to providing new predictions.

## Methods

There are many biological data sets that may be directly or indirectly related to PPIs. We tried to collect as many as possible for yeast and human.

### Feature set

For the various data sources, each of them has its own representative form. For example, protein sequence is in the form of a character string, which means the order of amino acids as they occur in a polypeptide chain. Gene expression data is usually a vector of expression values across multiple time points for a specific gene. Synthetic lethal data describes that a pair of genes having mutations together would affect the cells inviable or viable. So how could we combine these different forms of data together?

**Figure 1:**
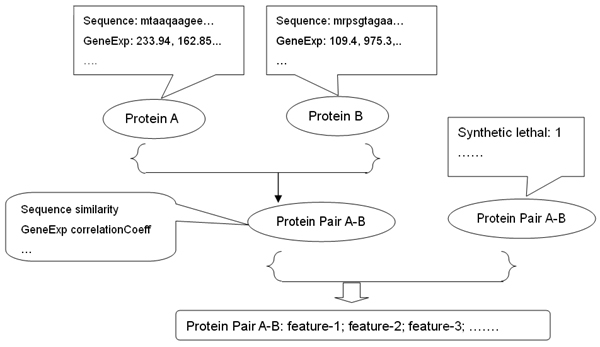
**Converting Data Sources to A Feature Vector Representing Each Protein-Protein Pair**. The process of combining biological sources and then converting them to feature vectors describing protein-protein pairs. For a gene/protein specific feature, we found a natural way to transform it to represent the protein-protein pair. For example, for gene expression data, we use the correlation coefficient as the feature for a protein-protein pair.

We present the converting process briefly in Figure 1. For each data set that represents a certain gene/protein's property, we figured out one natural way to calculate the similarity between two genes/proteins with respect to the specific evidence. For instance, for two proteins' sequence information, we use BlastP [27] sequence alignment E-value as one feature for this protein-protein pair from the protein sequence evidence. For other data sources, similar procedures were pursued to make the features for a protein pair. For data sets directly describing a protein/gene pair, we used them directly as features, like synthetic lethal evidence. Concatenating all these features together then gave us the feature vector describing a protein-protein pair.

#### Feature set for yeast

For yeast we collected a total of 162 feature attributes from 17 different data sources (Table 1). Three data sources are derived from the direct high-throughput yeast PPI data sets [1,2,5,6], with two from mass spectrometry and one from high-throughput yeast-two-hybrid screens. These evidence describe pair of proteins directly and thus are used as feature items in the feature vector. Six data sources represent each gene's functional annotations from [21,22]. The 'similarity' features derived from them represent how similar two proteins occur in the certain annotation space or from a specific function perspective. Four other different sources derived features that describe the similarity between two genes from sequence and structure perspectives. The remaining attributes are all based on indirect high-throughput experimental data. For example, this includes gene expression [8] correlations. All related data sources and how they were converted into features representing pair of proteins have been described in details previously in [15].

**Table 1:** Feature Set in Yeast.

**Expert**	**Feature Source**	**Size**	**Coverage (%)**
P	HMS-PCI MS	1	8.3
P	TAP MS	1	8.8
P	Yeast-2-Hybrid	1	3.9
F	GO Function	21	80.7
F	GO Process	33	76.1
F	GO Component	23	81.5
F	Essentiality	1	100
F	MIPS protein class	25	4.6
F	MIPS mutant phenotype	11	9.4
S	Gene fusion/cooccurence	1	100
S	Sequence similarity	1	100
S	Homology derived PPI	4	100
S	Domain interaction	1	100
E	Gene Expression	20	88.9
E	Protein Expression	1	42.8
E	Trans Factor Binding	16	98.0
E	Synthetic Lethal	1	7.6

Feature set derived for pairwise protein-protein interaction prediction in yeast. We used a total of 162 features from 17 different data sources. The first column lists the feature expert to which the feature source was assigned. We have designed a total of four experts: P, F, S and E (for definition see the 'Feature' section). The second column lists the name of the feature source. The third column lists the number of attributes from each source. The fourth column presents the average percentage of pairs for which information is available using this feature source. All related data sources and how they were converted into features have been described in details previous in: .

#### Feature set for human

For human we collected a total of 27 feature attributes from 8 different data sources (Table 2). Collecting data for human proteins is much harder than for yeast because several data sets that are available for yeast are not yet available for human and there exist much more human proteins than yeast.

**Table 2:** Feature Set in Human.

**Expert**	**Feature Source**	**Size**	**Coverage(%)**
F	GO Function	1	39.1
F	GO Component	1	36.3
F	GO Process	1	37.6
F	Tissue	1	57.1
E	Gene Expression	16	34.0
S	Sequence similarity	1	100
S	Yeast Homology PPI	5	100
S	Domain interaction	1	37.7

Feature set derived for pairwise protein-protein interaction prediction in human. We collected a total of 27 features from 8 different data sources. The first column lists the feature expert to which the feature source was attributed to. Unlike yeast, for human we had a total of three experts: F, E and S (for definition see the 'Feature' section). The second column lists the name of the feature source. The third column lists the number of attributes from each source. The fourth column presents the average percentage of pairs for which information is available using this feature source.

Similarly as in yeast, three kinds of 'similarity' features were derived from Gene Ontology (GO [21]) functional information, according to the two proteins' positions in the three ontology structures. In human, tissue distribution is an important property to describe a gene/protein. We used a binary feature to indicate if two proteins are expressed in the same human tissue or not. Gene expression features were derived from sixteen expression sets in NCBI Gene Expression Omnibus database [26]. Protein sequence alignment score was used as the another feature source. Homologous PPI was derived from the yeast protein-protein interaction datasets. Domain-domain interactions were derived based on the hypergeometric distribution and calculated for each candidate protein pair in the same way as in yeast feature set.

#### Feature properties

There are several feature properties we need to consider when designing computational approaches for the PPI prediction task. (1). Most biological datasets are *noisy *and contain many *missing *values. For example, in the Y2H derived features listed in Table 1, interactions involving membrane proteins are unreliable or missing. In both Table 1 and Table 2, the fourth column lists the average coverage of each feature source. As can be seen, different features have varying degrees of missing values. The average coverage of the 17 groups in Table 1 ranges from 3.9% for Y2H to over 88.9% for gene expression and 100% for sequence based features. (Coverage here means the percentage of pairs for which this feature is available). (2). The derived features are *heterogeneous*. Some features are categorical (for example, synthetic lethal [20]) while others are continuous (for example, mRNA co-expression [8]). In addition, some of them are highly correlated features (for example expression data from two different stress response experiments). (3). Finally, there is the issue of *weighting *these different data sources. Different protein pairs may benefit from using different feature sets in the prediction process. For every pair it would be useful for computational techniques to provide information about how features contribute to the classification predictions. For biologists who want to use these methods to build new hypotheses, integrating this information and their expert knowledge could assist lab experimental design.

### Feature experts

Overall, these biological data sources can be divided into four feature categories, which are referred to as feature experts in this paper:

1. Expert P: direct high-throughput experimental PPI data. This category contains those data sets that directly detected interaction relationships between proteins. They were derived through high-throughput biological experiments such as Y2H screens and mass spectrometry.

2. Expert E: indirect high-throughput data. This category includes those experimental data sources that were generated through high-throughput techniques and represent certain aspects of genes/proteins other than PPI relationship, such as gene expression and protein-DNA binding.

3. Expert S: sequence based data sources. This category includes those features that represent how similar two proteins are based on sequence or structure information. For example, this expert includes domain information and gene fusion data.

4. Expert F: functional properties of proteins. This category contains information about how similar two proteins are in terms of functional annotations such as biological process, protein localization, protein class, and essentiality.

Note that in human there are only two very small Y2H data sets [4,3] available. We therefore currently do not have a 'P' feature expert for human data. As more data sets become available, this feature expert can be generated for human as well.

After splitting, the features within experts are derived from similar data sources and are roughly *homogeneous *when compared with each other. Usually biologists could give opinions and make comparisons on general categories of biological evidence. Thus, it would also be useful for computational methods to provide automatic information about how several feature categories (experts) contribute to every predicted interaction pair. The derived computational importance together with biologists' expert knowledge could assist the further prediction analysis and the design of lab PPI experiments. In this work, we divided features into four experts. Apparently, the number of experts to be split into could be different. The splitting depends on the need of the application and the analysis ability of the biologists who would validate the predictions.

### Mixture-of-Feature-Experts (MFE)

Using our experts setting, features are grouped into four (for yeast) or three (for human PPIs) categories. While the features are heterogeneous overall, within feature experts, attributes are roughly homogeneous and are derived from similar data sources. Our main intuition in using the expert-based structure is to investigate the relationship between these homogeneous feature groups in terms of predicting PPIs and to compare the importance of experts contributing to each prediction. This provides a principled way for selecting informative feature types during the prediction process.

**Figure 2:**
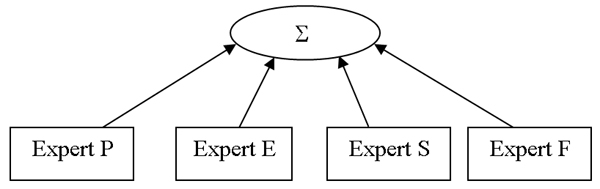
**Mixture of Four Feature Experts in Yeast**. Graphical representation of the Mixture-of-Feature-Experts method (MFE) for yeast. Table 1 lists the features used by each of the four experts. For definition of P, F, S, E experts, see details in the 'Feature' section.

We design a method called Mixture-of-Feature-Experts (MFE) to achieve the above computational properties. As Figure 2 shows, our framework can be viewed as a single layer tree, with feature experts at the leaves. Each expert uses one of the dataset groups to predict PPIs. A root gate is used to integrate predictions from multiple feature experts. The weights assigned to each of the experts by the root gate depends on the input set for a given pair. Intuitively, this framework is analogous to the following process: each feature expert gives their opinion about how likely the investigated pair interacts and then the gate creates a final decision by the weighted sum of the experts' predictions. Moreover, these weights are local and specific to the current example pair.

In the following sections, *X *describes the input feature vector variable and *Y *represents the target output variable. Input variable *X *represents *d*-dimensional feature vectors built from features in Table 1 or Table 2. Target variable *Y *∈ {-1, 1} means whether a protein pair interacts (1) or not (-1).

**Figure 3:**
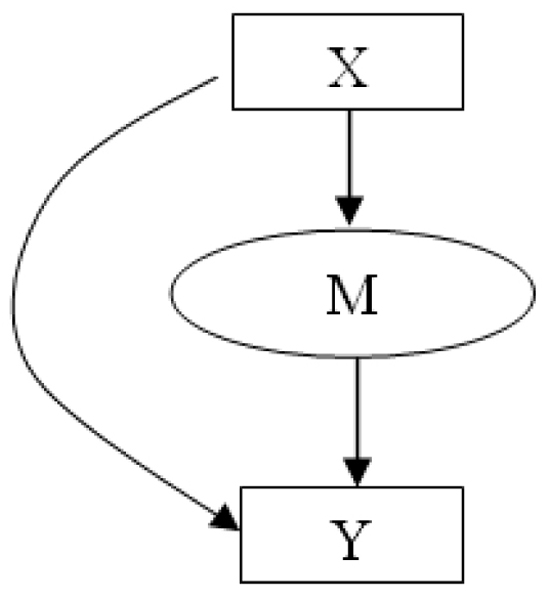
**Graphical Model View of Mixture-of-Experts (ME) Method**. A graphical model view of the Mixture-of-Experts (ME) method. The target variable *Y *is dependent on the input vector *X *and the multinomial random variable *M*. *P*(*M*|*X*) is modeled by the gate while *P*(*Y*|*X*, *M*) is modeled by the experts.

Given our feature experts setting, the conditional probability of the target variable *Y *given the input variable *X *could be written as:

$$P(Y|X)=\sum_{M}P(M|X)P(Y|X,M)$$

where *M *is a set of *hidden *data and indicates which expert was responsible for generating each example data pair. Having *I *experts, *M *is a *I*-dimensional indicator vector variable. That is, all entries in *M *are 0 except for one of the entries which is set to 1. The sum is over all configuration of variable *M*. In other words, target class label *Y *is dependent on the input data *X *and the choice of expert *M*. The choice of *M *is also dependent on the input *X*. *P*(*M*|*X*) is modeled using the root gate, while *P*(*Y*|*M*, *X*) is modeled by each feature expert in our framework. The graphical model view of MFE method is illustrated in Figure 3. This Bayesian network structure expresses that the target variable *Y *is dependent on the input vector variable *X *and the multinomial random variable *M*. It is essentially a modification of the probabilistic *Mixture-of-Experts *(ME) model [32].

Using a training set including *N *examples, the *n*-th example pair is described using (*x*^(*n*)^, *y*^(*n*)^). For *n *= 1 to *N*, each data example (*x*^(*n*)^, *y*^(*n*)^) has a corresponding vector *m*^(*n*)^. The dimension of vector *m*^(*n*) ^is equal to the number of feature experts: *I *(*I *= 4 for yeast and *I *= 3 for human). With *i *= 1 to *I*, *n *= 1 to *N*, each entry of this vector $m_{i}^{\left(n\right)}$ is as following:

$$m_{i}^{\left(n\right)}=\{\begin{array}{ll} 1, & \text{if using feature expert }i\text{ for example }n\\ 0, & \text{otherwise}. \end{array}$$

Thus, based on Equation (1) the conditional probability *P*(*y*^(*n*)^|*x*^(*n*)^) is formulated specifically as:

$$P(y^{\left(n\right)}|x^{\left(n\right)})=\sum_{i=1}^{I}P(m_{i}^{\left(n\right)}=1|x^{\left(n\right)},v)P(y^{\left(n\right)}|x^{\left(n\right)},m_{i}^{\left(n\right)}=1,\omega_{i})$$

where *w*_*i *_are the model parameters used for feature expert *i *and *v *contains the model parameters used for the gate.

In general each expert can take any form such that the expected value of their probability density is consistent with the form of the problem. In this work, we use binary logistic regression for each of the feature experts. For the *i*-th expert (*i *= 1...*I*) we write:

$$P(y^{\left(n\right)}|x^{\left(n\right)},m_{i}^{\left(n\right)}=1,\omega_{i})=\frac{1}{1+exp(-y^{\left(n\right)}(w_{i}^{T}x^{\left(n\right)}))}$$

Similarly, the root gate can take any functional form that is consistent with a probability distribution. For instance [32] used multinomial logit models for the gates. Here, we extend the binary logistic regression to model the multinomial probability distribution of variable *M *through voting. This is analogous to using the one-versus-all strategy to transform a *I*-class classification into *I *binary logistic regression problem [34]. First binary logistic regression model is run once for each output branch of the root gate. Next, modified probability weights are calculated for each branch by combining all the branch models. Each branch of the root gate controls the weighting of a certain feature expert in our work. For the *i*-th branch (*i *= 1...*I *for our gate) *v*_*i *_represent the logistic regression parameters for this branch and variable *C*_*i *_represents the binomial probability distribution from this branch. Thus,

$$P(c_{i}^{\left(n\right)}=1)=\frac{1}{1+exp(-(v_{i}^{T}x^{\left(n\right)}))}$$

then by normalizing over all branches, we get the multinomial probability distribution of variable M as below:

$$P(m_{i}^{\left(n\right)}=1|x^{\left(n\right)},v)=\frac{P(c_{i}^{\left(n\right)}=1)}{\sum_{j=1}^{I}P(c_{j}^{\left(n\right)}=1)}$$

This means that *P*($m_{i}^{\left(n\right)}$ = 1|*x*^(*n*)^, *v*) depends on the input attributes (*x*^(*n*)^) and it represents the gate weight for expert *i *when predicting the *n*-th pair. In all of the above logistic regression steps, we apply ridge estimators to infer stable regularized parameters.

In summary within our feature experts framework the interaction prediction from MFE is a weighted sum of the opinions from each feature expert. The weights assigned to each expert are controlled by the input feature values as well as by the feature experts.

### Expectation-Maximization (EM)

Based on the probabilistic model in Equation (1), learning in MFE architecture is treated as a maximum likelihood problem. The model parameters include the gate parameters *v *and the expert parameters *ω*_*i*_.

We compute the log likelihood by taking the logarithm of the products of *P*(*y*^(*n*)^|*x*^(*n*)^) as follows,

$$ll=\sum_{n=1}^{N}log(\sum_{i=1}^{I}P(m_{i}^{\left(n\right)}=1|x^{\left(n\right)},v)P(y^{\left(n\right)}|x^{\left(n\right)},m_{i}^{\left(n\right)}=1,\omega_{i}))$$

In the following we use Θ as the set of all the parameters including both experts and gate parameters. Jordan and Jacobs [31] have proposed an expectation-maximization (EM) algorithm for adjusting parameters in ME architecture. The EM algorithm is an iterative approach for maximum likelihood estimation (MLE). Each iteration of an EM algorithm consists of two steps, the E-step and the M-step. For the *t*-th epoch, model parameters are represented as Θ^*t*^.

In the E-step we compute the posterior probability $h_{i}^{\left(n\right)}$ using Equation (8). $h_{i}^{\left(n\right)}$ represents the posterior weight for expert *i *in predicting pair *n *once both the input and the target output are known. $h_{i}^{\left(n\right)}$ is derived using Bayes rule:

$$h_{i}^{\left(n\right)}=P(m_{i}^{\left(n\right)}=1|x^{\left(n\right)},y^{\left(n\right)},\Theta^{t})$$

$$=\frac{P(m_{i}^{\left(n\right)}=1|x^{\left(n\right)},\Theta^{t})P(y^{\left(n\right)}|x^{\left(n\right)},m_{i}^{\left(n\right)}=1,\Theta^{t})}{P(y^{\left(n\right)}|x^{\left(n\right)},\Theta^{t})}$$

$$=\frac{P(m_{i}^{\left(n\right)}=1|x^{\left(n\right)},v^{t})P(y^{\left(n\right)}|x^{\left(n\right)},m_{i}^{\left(n\right)}=1,\omega_{i}^{t})}{\sum_{j=1}^{I}P(m_{j}^{\left(n\right)}=1|x^{\left(n\right)},v^{t})P(y^{\left(n\right)}|x^{\left(n\right)},m_{j}^{\left(n\right)}=1,\omega_{j}^{t})}$$

By decomposition of the expected complete data-likelihood, the M-step reduces to separate maximization problems [31,32], one for each expert and gate. In our MFE framework it solves the following maximization problems: for each expert,

$$\omega_{i}^{t+1}=argmax_{\omega_{i}}\sum_{n=1}^{N}h_{i}^{\left(n\right)}log(P(y^{\left(n\right)}|x^{\left(n\right)},m_{i}^{\left(n\right)}=1,\omega_{i}))$$

and for the root gate,

$$v^{t+1}=argmax_{v}\sum_{n=1}^{N}\sum_{j=1}^{I}h_{j}^{\left(n\right)}log(P(m_{j}^{\left(n\right)}=1|x^{\left(n\right)},v))$$

Each of these maximization problems are themselves maximum likelihood problems [31,32]. Equation (11) is simply the general form of a weighted maximum likelihood problem in the probability density *P*(*y*^(*n*)^|*x*^(*n*)^, $m_{i}^{\left(n\right)}$ = 1, *ω*_*i*_). Given our expert choice, the log likelihood in Equation (11) is a weighted log likelihood (weighted by $h_{i}^{\left(n\right)}$) for the logistic regression model. An efficient algorithm known as iteratively reweighted least-squares (IRLS) is available to solve this maximum likelihood task [31].

Equation (12) involves maximizing the cross-entropy between the posterior probability $h_{j}^{\left(n\right)}$ and the prior probability *P*($m_{j}^{\left(n\right)}$ = 1|*x*^(*n*)^, *v*). This cross-entropy is the log likelihood associated with a multinomial logistic gate model in which the $h_{j}^{\left(n\right)}$ could be treated as output observations. Thus the maximization in Equation (12) is a maximum likelihood problem for a generalized linear model and can also be solved using IRLS technique.

Overall the EM algorithm could be summarized as the following iterative process:

1. For each data pair (*x*^(*n*)^, *y*^(*n*)^), compute the posterior probability $h_{i}^{\left(n\right)}$ using the current values of the parameters.

2. For each expert *i*, solve a maximization problem in Equation (11) with observation ${\left\{x^{\left(n\right)},y^{\left(n\right)}\right\}}_{n=1}^{N}$ and observation weights ${\left\{h_{i}^{\left(n\right)}\right\}}_{n=1}^{N}$.

3. For the root gate, solve the maximization problem in Equation (12) with observation ${\left\{x^{\left(n\right)},y^{\left(n\right)}\right\}}_{n=1}^{N}$ and observation weights ${\left\{{\left\{h_{i}^{\left(n\right)}\right\}}_{n=1}^{N}\right\}}_{i=1}^{I}$.

4. Iterate by using the updated parameter values until a termination criterium is satisfied.

### Handling the missing feature value problem

As pointed out, biological datasets contain many missing values and this problem is an important obstacle in achieving significant improvements in prediction performance.

The simplest approach to handle the missing feature items is to fill those missing entries by certain values. For example, for a real-valued feature the filled value could be the mean of the feature column or for a categorical feature we could use the most common value. In the following sections we use the term 'MFE-FM' to represent the MFE method while using mean estimates for missing values (MFE-FM: mixture of feature experts with missing values filled).

We apply a more principled strategy to handle missing feature values. Specifically, for each feature that has low feature coverage, this strategy add an extra feature column to represent the feature availability. For *d *= 1...*D *(*D *= 162 for yeast and *D *= 27 for human), *X*_*d *_represents the *d*-th feature column and *g*(*X*_*d*_) describes the ratio of missing cases for feature *X*_*d*_. If *g*(*X*_*d*_) is larger than a predefined ratio, we add a new, binary, feature column *X*_(*D*+1) _to represent the availability of feature *X*_*d*_. That is, if for an example pair the feature *X*_*d *_is missing, this new feature *X*_(*D*+1) _would be set to 0. Otherwise it would be set to 1. The method now uses this new feature and can learn different parameters for observed and estimated features. Totally if there are *p *original feature columns that have new feature columns added, the final feature vector then grows to be *D *+ *p *dimensional. While this strategy increases the size of our feature set, it is still very small (~200 for yeast and ~50 for human) compared to the total number of protein pairs (~18 M for yeast and ~4000 M for human).

In our MFE framework, since the weighting depends on the input features, by this adding features strategy our classifiers can use the present/absent information to modify the weights of different feature experts. Similarly this strategy could also improve the classifiers used by each feature expert. In the following sections the term 'MFE' means the MFE method when using this added extra features strategy.

## Results

We first discuss the reference sets and evaluation strategies used in performance comparisons. Next we present results for comparing the MFE method to several popular classifiers for predicting protein interaction pairs in yeast and human.

### Reference set (gold standard set)

Any classification algorithm requires a training set. In our work for the positive set, there are a small number of interacting protein pairs that have been reliably determined by small-scale laboratory experiments. This set serves as our positive standard for this learning problem. For yeast, ~2900 interacting protein pairs were extracted from the database of interacting proteins (DIP) [23]. For human, ~15,000 protein-protein interaction pairs were extracted from the Human protein reference database (HPRD) [24]. Both sets were filtered to exclude self-interactions.

Unlike positive interactions, it is rare to find a confirmed report on non-interacting pairs. Considering the small fraction of interacting pairs in the total set of potential protein pairs we use a random set of protein pairs, excluding those interacting pairs that are known, as the negative set. In yeast, it is estimated that roughly only 1 in about 600 possible pairs actually interacts [14]. In human, this ratio is even smaller, roughly 1 in several thousands of possible pairs is estimated to interact. Thus, over 99.8% of our random data is indeed non-interacting, which is probably better than the accuracy of most training data.

Combining the positive and negative PPI sets, a reference set (also referred to as gold standard set) is then constructed for use as training/testing sets when applying learning methods.

### Evaluation strategy

Based on the reference set, we use the following two measures to evaluate the performance of our predictions, Precision vs. Recall curves and AUC scores (area and partial areas under the Receiver Operator Characteristic curve).

In Precision vs. Recall curves, Precision refers to the fraction of interacting pairs predicted by the classifier that are truly interacting (true positives). Recall measures how many of the known pairs of interacting proteins have been identified by the learning model. The Precision vs. Recall curve is then plotted for different cutoffs on the predicted score.

Receiver Operator Characteristic (ROC) curves plot the true positive rate against the false positive rate for different cut-off values of the predicted score. It measures the trade-off between sensitivity and specificity. The area under the ROC curve (AUC) is commonly used as a summary measure of diagnostic accuracy. It can take values from 0.0 to 1.0. In some cases, rather than looking at the area under the entire ROC curve, it is more informative to only consider the area under a portion of the curve. In our prediction task, we are predominantly concerned with the detection performance of our models under conditions where the false positive rate is low. For example, R50 is a partial AUC score that measures the area under the ROC curve until reaching 50 negative predictions. Similarly R100 is the partial AUC score when reaching 100 negative predictions.

### Performance Comparison

To measure the ability of the MFE method to predict PPIs, we compared it with four other popular classifiers that have been suggested in the past for this task: Logistic Regression (LR), Naïve Bayes (NB), Support Vector Machines (SVM) and Random Forest (RF). Our MFE method is implemented using Matlab. Standard toolkits are used for the other methods. Specifically, The SVMlight toolkit was used for SVM [30]. Logistic Regression and Naive Bayes were obtained from the WEKA machine learning tool box [29]. Random Forest was from the Berkeley RF package [28]. The input feature vectors to these methods are exactly the vectors from Table 1 or Table 2 with missing values filled.

All comparisons were based on the following training and testing procedures. In yeast, we randomly sampled a training set containing ~30,000 protein pairs to learn the decision model. Then we sampled a test set (another ~30,000 pairs) from the remaining protein pairs, and used the trained model to evaluate the performance of the classifiers. The above steps were repeated 10 times for each classifier and average values are reported. Similar procedures were pursued in human where the training and the testing sets included ~80,000 examples. For each evaluated classifier, parameter optimization was carried out in all cases in identical train-test fashion.

**Figure 4:**
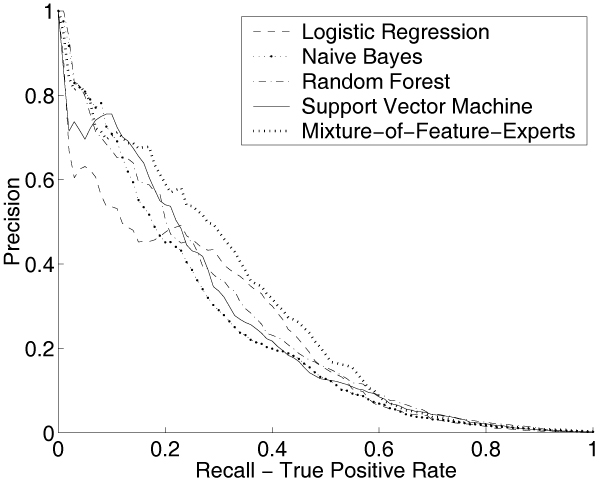
**Performance Comparison in Yeast**. Average Precision vs. Recall curves when comparing MFE method with four other classifiers (LR/NB/RF/SVM) for PPI prediction in yeast. LR: Logistic regression; NB: Naive Bayes; RF: Random Forest; SVM: Support Vector Machine; MFE: Mixture-of-Feature-Experts. The MFE curve dominates the curves for the other four methods in most of the recall ranges.

**Figure 5:**
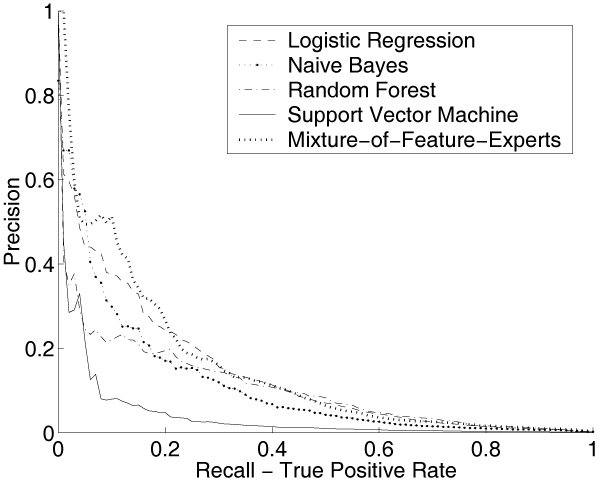
**Performance Comparison in Human**. Average Precision vs. Recall curves when comparing MFE with four other classifiers (LR/NB/RF/SVM) for PPI prediction in human. LR: Logistic regression; NB: Naive Bayes; RF: Random Forest; SVM: Support Vector Machine; MFE: Mixture-of-Feature-Experts. Again, the MFE curve dominates the other four curves for most of the low recall values.

Based on the estimated ratio of interacting versus non-interacting pairs in yeast and human, we have roughly ~50 to ~100 positive PPIs in each test run. For the training set, we up-sampled the positive examples in a pre-processing step, which resulted in roughly ~800 positive examples for each training run in human and roughly ~300 positive pairs for each yeast training. This sampling strategy reduces the problem of too few positive examples in the training set without affecting the performance significantly [33]. Figure 4 plots the average precision versus recall curves of these five different methods for the yeast PPIs prediction and Figure 5 is for human. In both figures, the curves derived from MFE approach dominate the other four methods in most of the low recall ranges.

Table 3 lists the average AUC score and partial AUC scores for the yeast PPI evaluation. The standard derivations for each score estimation are also listed in the table. MFE scores are highlighted and it clearly achieves better AUC/R50/R100 scores compared to the other methods. For instance, MFE improves the R50 score by ~7% when compared to the other classifiers tested. Table 4 lists the scores for the human data set. Similarly as for yeast, the MFE method achieves better results. For example, MFE achieves ~10% improvement in R50 score compared to the other classifiers used. Thus the MFE method achieves the best results for all criteria tested.

**Table 3:** Average AUC and Partial AUC scores in Yeast.

**Method**	**AUC mean**	AUC std	**R50 mean**	R50 std	**R100 mean**	R100 std
LR	0.8823	0.033	0.2866	0.070	0.3546	0.073
NB	0.9349	0.015	0.2486	0.047	0.3135	0.062
RF	0.9321	0.014	0.2688	0.048	0.3434	0.049
SVM	0.9159	0.024	0.2585	0.063	0.3262	0.067
MFE	**0.9463**	0.013	**0.3080**	0.078	**0.3799**	0.077
MFE-FM	0.9220	0.021	0.2918	0.061	0.3738	0.058

Average AUC and partial AUC scores for six classification methods for PPI prediction in yeast. LR: Logistic regression; NB: Naive Bayes; RF: Random Forest; SVM: Support Vector Machine; MFE: Mixture-of-Feature-Experts; MFE-FM: Mixture-of-Feature-Experts with missing features filled. Average AUC and partial AUC scores are reported and the standard derivations for each score estimation are also listed in the table. MFE scores are highlighted and it clearly achieves better AUC/R50/R100 scores compared to the other five.

**Table 4:** Average AUC and Partial AUC scores in Human.

**Method**	**AUC mean**	AUC std	**R50 mean**	R50 std	**R100 mean**	R100 std
LR	0.9419	0.020	0.1148	0.031	0.1684	0.031
NB	0.9389	0.003	0.0964	0.031	0.1356	0.035
RF	0.9427	0.009	0.0740	0.025	0.1263	0.030
SVM	0.7645	0.091	0.0455	0.028	0.0589	0.040
MFE	**0.9608**	0.007	**0.1341**	0.023	**0.1759**	0.027
MFE-FM	0.9384	0.018	0.1297	0.023	0.1713	0.025

Average AUC and partial AUC scores for six classification methods for PPI prediction in human. LR: Logistic regression; NB: Naive Bayes; RF: Random Forest; SVM: Support Vector Machine; MFE: Mixture-of-Feature-Experts; MFE-FM: Mixture-of-Feature-Experts with missing values filled. Average AUC and partial AUC scores are reported and the standard derivations for each score estimation are also listed in the table. MFE scores are highlighted and it again achieves better AUC/R50/R100 scores compared to the other five classifiers.

The last two rows of Table 3, list the AUC and partial AUC scores of MFE-FM and MFE methods in yeast. MFE clearly achieves better performance compared to MFE-FM (~3% increase in R50 score). This means that by explicitly indicating the availability of feature attributes our method improves the classification outcome. Similar conclusions could be drawn for human as shown in Table 4.

The methodology we propose, of feature experts, is very general. As discussed in the 'Methods' section, the number of feature experts the heterogeneous data sets are split into could be different. The splitting essentially depends on the need of the application and the preference of the biologists who would analyze and/or validate the predictions. At the limit case, we can assign each feature to an individual expert. To test this we carried out one new experiment for the human prediction task treating every feature as its own expert. As the results (supporting Figure 1[36]) indicate, this does not improve the performance of the algorithm, perhaps because it leads to overfitting of the parameters.

## Discussion

Biologically, it is of particular interest to identify the extent to which heterogeneous data sources carry information about protein interactions. An analysis of the contribution of different features can also help uncover relationships between different data sources that are not directly apparent.

Analysis of feature importance is important on the global scale as well as for the prediction and analysis of specific protein pairs. We therefore ask the following questions: (1) How do the different features affect PPI prediction performance overall? and (2) How do the different features contribute differently for each example pair? We have explored these two questions using the yeast results.

### Global feature importance

To control data collection costs, it is important to select only informative data types globally. Once informative data types are identified, one does not need to use unnecessary data sets when solving similar network inference problems for other sets of proteins or for other organisms. This can significantly speed up prediction of PPIs in new species, as well as when updating predictions on model species such as yeast and human with new data sources.

To identify overall feature importance among our feature experts, we remove feature experts one by one, and run the MFE methods on the remaining three experts. We then examine how the performance changes. Table 5 lists the score changes of R50 and AUC after removing the experts one by one. The less the score changes the less important is the feature expert. We found that removing the sequence expert 'S' had the least impact on both scores. The indirect high-throughput data expert 'E' ranked second from the bottom in the prediction of yeast PPI's.

**Table 5:** Global Feature Expert Importance (by MFE) in Yeast.

	MFE R50	R50 DROP	MFE AUC	AUC DROP
P	0.2310	0.0770	0.9244	0.0219
F	0.2609	0.0471	0.8821	0.0642
S	0.3191	-0.0111	0.9459	0.0004
E	0.3022	0.0058	0.9323	0.0140

Full	0.3080		0.9463	

Global feature expert importance can be measured by the decrease in AUC and R50 scores when removing the expert in the MFE method. The first column lists the four feature experts. The second and fourth column list the R50 and AUC scores when applying MFE while only using the remaining three experts. The third and fifth column list the changes between these R50 and AUC scores and the full experts version. For definition of P, F, S, E experts, see details in the 'Feature' section and Table 1.

It is surprising that removing expert 'E' (which contains mostly microarray expression data) does not hurt performance much. This is seemingly in contradiction to previous estimations in which tree based feature ranking methods ranked gene expression features very highly [15]. Note that, when the feature sets are not grouped, the wide availability of gene expression data and its high coverage may result in an increased use of this feature, even though it may lead to overfitting. As our results suggest, splitting the data into more homogeneous groups (feature experts here) may help increase the prediction accuracy by decreasing its reliance on these high throughput data sources.

### Feature importance for specific protein pairs

For each predicted pair it would be useful for computational techniques to provide information about which features contributed to the predictions for that pair. Our MFE method naturally reveals how each feature category contributes to the interaction predictions. The posterior probability from Equation (8) could be treated as the level of contribution from each expert to the final prediction. Then for a specific candidate protein pair, these values could give a detailed description about how each expert contributes to the integrated prediction.

**Figure 6:**
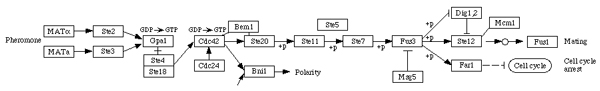
**Yeast Pheromone Response Pathway**. The yeast pheromone response pathway. This figure is from the KEGG [25] database.

To demonstrate the utility of this unique capability of the MFE method to reveal feature importance in specific predictions, we investigated a specific yeast pathway; the yeast pheromone response. For this pathway we compare the contribution of different experts in the known and predicted interacting pairs. Figure 6 presents the known interactions in this pathway as determined by the KEGG database [25]. In this pathway the yeast mating factors MAT alpha/a bind to their cognate membrane receptors Ste2/3, members of the G protein coupled receptor family. Subsequent binding and activation of the G protein induces a MAP kinase signaling pathway via G protein activation [35].

We selected 25 proteins that are known to participate in this pathway and applied the MFE algorithm to classify the 300 (25*24/2) potentially interacting pairs. The training set included 500 positive pairs and 50000 negative (random) pairs. None of these pairs contained any of the known 25 proteins in this pathway. The positive versus negative ratio in this set is roughly the same as the ratio we used for the performance comparisons. We determined a prediction threshold using the training set. 51 of the 300 pairs had scores above the threshold and were thus predicted to be interacting. Among them, 33 interactions (64.7%) had been experimentally validated. The remaining 18 pairs are new predictions.

**Figure 7:**
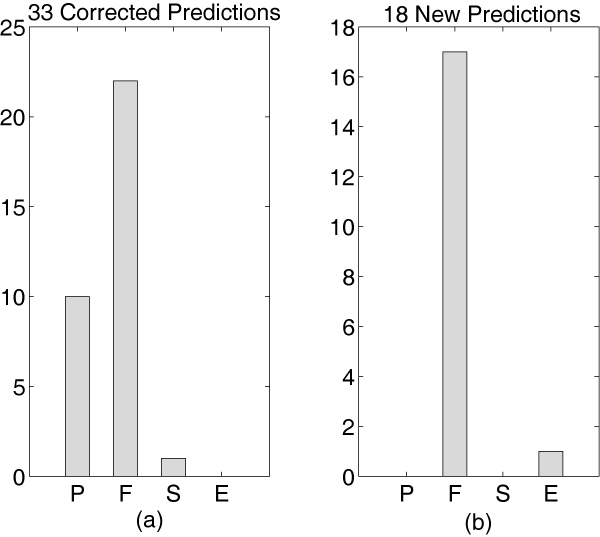
**Pair Feature Importance Analysis**. Distribution of highest scoring experts for the yeast pheromone response pathway validation. For definition of P, F, S, E experts, see details in Table 1 and the 'Feature' section. (a) shows the frequency at which each of the four experts had the maximal score for the 33 known interacting pairs. (b) shows the frequency at which each of the four experts had the maximal score for the 18 new predictions.

Figure 7(a) shows the frequency at which each of the four experts showed maximal contributions among validated pairs. In line with biological intuition, the direct high-throughput evidence (expert P) and functional databases (expert F) are the predominant experts in the correct predictions. Figure 7(b) shows that the majority of the 18 new predictions are based on recommendations by expert F. Based on the reliability of expert F in making correct predictions, this result indicates that the majority of the new predictions may turn out to be correct, once experimentally tested.

## Conclusion

One of the most important goals of computational PPI predictions is to suggest biological hypotheses regarding unexplored new interactions that are testable with subsequent experimentation. Among high scoring predictions, the most interesting ones can be chosen by an individual investigators using intuition and specialized knowledge.

This paper addresses two important problems for the PPI prediction task. First, previous classification methods estimate a set of parameters that are used for all input pairs. However, the biological datasets used contain many missing values and highly correlated features. Thus, different samples may benefit from using different feature sets. The second problem is that biologists who want to use these methods to select experiments cannot easily determine which of the features contributed to the resulting prediction. Since different researchers may have different opinions regarding the reliability of the various feature sources, it is useful if the method can indicate, for every pair, which feature contributes the most to the classification result.

In this paper we propose a Mixture-of-Feature-Experts (MFE) approach to address the above two challenges when predicting protein-protein interactions. Diverse high-throughput biological datasets are split into homogeneous feature experts. Each expert uses a subset of the data to predict protein interactions and expert predictions are combined such that the weight of each expert depends on the input data for the predicted protein pair. This method is useful for overcoming problems in achieving high prediction performance arising due to missing values which are a major issue when analyzing biological datasets. In addition, the weights can be used by biologists to determine confidence in the prediction for each pair. We have shown that this algorithm improves upon previous methods suggested in yeast and human for this task. Extensions of this approach to other species are straight forward when more information becomes available.

We believe that as the prediction task becomes harder (for example, when analyzing human HIV related interactions) the need for methods that can accommodate high levels of missing values and are directly interpretable by biologists increases. The next step will be to apply our method to interaction prediction tasks related to important types of human proteins where missing values and the small number of positive examples are major obstacles in obtaining of an accurate protein interaction map.

## List of abbreviations used

• Y2H: Yeast-Two-Hybrids;

• PPIs: Protein-Protein Interactions;

• MFE: Mixture of Feature Experts;

• MFE-FM: Mixture-of-Feature-Experts with Missing Values Filled;

• ME: Mixture of Experts;

• EM: Expectation Maximization;

• LR: Logistic regression;

• NB: Naive Bayes;

• RF: Random Forest;

• SVM: Support Vector Machine;

## Competing interests

The authors declare that they have no competing interests.

## Authors' contributions

QYJ carried out the feature experts method designs, performed the methods implementation and analysis and drafted the manuscript. JKS participated in the design and analysis of the study and helped to revise the manuscript. ZBJ participated in the method design and analysis and helped to draft the manuscript. All authors read and approved the final manuscript.
